# Clinical and Muscle Imaging Findings in 14 Mainland Chinese Patients with Oculopharyngodistal Myopathy

**DOI:** 10.1371/journal.pone.0128629

**Published:** 2015-06-03

**Authors:** Juan Zhao, Jing Liu, Jiangxi Xiao, Jing Du, Chengli Que, Xin Shi, Wei Liang, Weiping Sun, Wei Zhang, He Lv, Yun Yuan, Zhaoxia Wang

**Affiliations:** 1 Department of Neurology, Peking University First Hospital, Beijing, China; 2 Department of Radiology, Peking University First Hospital, Beijing, China; 3 Department of Pneumology, Peking University First Hospital, Beijing, China; RIKEN Advanced Science Institute, JAPAN

## Abstract

Oculopharyngodistal myopathy (OPDM) is an extremely rare, adult-onset hereditary muscular disease characterized by progressive external ocular, pharyngeal, and distal muscle weakness and myopathological rimmed vacuole changes. The causative gene is currently unknown; therefore, diagnosis of OPDM is based on clinical and histopathological features and genetic exclusion of similar conditions. Moreover, variable manifestations of this disorder are reported in terms of muscle involvement and severity. We present the clinical profile and magnetic resonance imaging (MRI) changes of lower limb muscles in 14 mainland Chinese patients with OPDM, emphasizing the role of muscle MRI in disease identification and differential diagnosis. The patients came from 10 unrelated families and presented with progressive external ocular, laryngopharyngeal, facial, distal limb muscle weakness that had been present since early adulthood. Serum creatine kinase was mildly to moderately elevated. Electromyography revealed myogenic changes with inconsistent myotonic discharge. The respiratory function test revealed subclinical respiratory muscle involvement. Myopathological findings showed rimmed vacuoles with varying degrees of muscular dystrophic changes. All known genes responsible for distal and myofibrillar myopathies, vacuolar myopathies, and muscular dystrophies were excluded by PCR or targeted next-generation sequencing. Muscle MRI revealed that the distal lower legs had more severe fatty replacement than the thigh muscles. Serious involvement of the soleus and long head of the biceps femoris was observed in all patients, whereas the popliteus, gracilis and short head of biceps femoris were almost completely spared, even in advanced stages. Not only does our study widen the spectrum of OPDM in China, but it also demonstrates that OPDM has a specific pattern of muscle involvement that may provide valuable information for its differential diagnosis and show further evidence supporting the conclusion that OPDM is a unique disease phenotype.

## Introduction

Oculopharyngodistal myopathy (OPDM; MIM 164310) is a very rare adult-onset neuromuscular disease characterized by slowly progressive blepharoptosis, ophthalmoparesis, facial and bulbar muscle weakness, and distal muscle weakness [1,2]. Muscle biopsies exhibit muscular dystrophic changes with rimmed vacuoles [3]. Since its first description by Sartoyoshi in 1977 [4], OPDM has only been observed in approximately 80 patients from 24 unrelated families from Turkey, Japan, the Netherlands, Italy, the United States and China [1–9]. OPDM is considered to be a clinically and genetically distinct myopathy, although its genetic defect has yet to be identified [1,2]. Therefore, current diagnoses of OPDM are primarily based on clinical features, histopathological findings, and genetic exclusion of similar conditions.

There is considerable clinical and myopathological overlap between OPDM and a wide spectrum of other muscular disorders, such as oculopharyngeal muscular dystrophy (OPMD), myotonic dystrophy 1 (DM1), distal myopathy with rimmed vacuoles, and myofibrillar myopathy [5,6,10]. OPMD is an autosomal dominant late-onset disorder characterized by progressive ptosis, dysphagia, and proximal muscle weakness. The genetic basis of OPMD has been identified as an abnormal trinucleotide (GCN) repeat expansion encoding the polyalanine tract in exon 1 of the polyadenylate-binding protein nuclear 1 gene (PABPN1) [11]. DM1 is an adult-onset autosomal dominant disorder caused by a heterozygous trinucleotide repeat expansion (CTG)n in the 3-prime untranslated region of the dystrophia myotonica protein kinase gene (DMPK), which is characterized mainly by myotonia, distal muscle weakness and multiple extramuscular system involvement, including cataracts, hypogonadism, frontal balding, and electrocardiogram (ECG) changes [12]. Many other distal myopathies also share the clinical features of preferentially distal muscle weakness and myopathological changes of rimmed vacuoles with OPDM. Advanced molecular genetic techniques have clarified the gene defect in approximately 20 distinct entities in the group of distal myopathies [10].

Recent studies have suggested that there might be differences in clinical manifestations among OPDM patients from different populations [1,2,5–8]. Therefore, additional OPDM cases and differential diagnostic markers would enable us to gain additional insight into this disease. Recently, muscle magnetic resonance imaging (MRI) has become a useful tool for clarification of neuromuscular disorders, and an increasing amount of evidence demonstrates that the MRI pattern of muscle involvement can be consistent in patients that harbor the same genetic defect, despite exhibiting clinical variability [10,13–15]. Herein, we report a comprehensive clinical evaluation and molecular genetic study of a group of mainland Chinese OPDM patients. Additionally, we investigated whether the MRI pattern of muscle involvement was consistent and specific to OPDM.

## Materials and Methods

### Ethics Statement

This study was conducted according to the principles of the Declaration of Helsinki and approved by the Ethical Committee of Peking University First Hospital. All patients provided written informed consent for the collection of samples and subsequent analyses.

### Patients

Fourteen OPDM patients (all Han Chinese; seven males and seven females) from 10 unrelated families were recruited at the Department of Neurology, Peking University First Hospital from 2005 to 2014. One family has been reported previously [3]. Their clinical records were retrospectively collected and analyzed. All of the patients were interviewed and examined by at least two experienced neurologists (W.Z., Y.Y., or Z.W.).

### Laboratory examination

The patients underwent comprehensive clinical evaluations, including ECG and ultrasonic cardiogram (n = 13), electromyography (n = 14), fiber optic endoscopic examination of swallowing (n = 6), and respiratory function evaluation (n = 10). All examinations were performed according to standard procedures. The parameters of the respiratory function evaluation included the ratio of the measured value of forced vital capacity to the predicted value (FVC), ratio of the measured value of the forced expiratory volume at the first second to the predicted value (FEV1) in the sitting and supine positions, FEV1/FVC ratio in the sitting and supine position, maximal inspiratory pressure (MIP), and maximal inspiratory pressure (MEP).

### Muscle imaging

Ten patients (cases 2, 3, 5–7, and 10–14) underwent muscle MRI examinations. Patients 3 and 12 had an additional MRI of the thigh muscle 1 year later, and patient 12 had a second imaging examination 2 years later. Muscle MRI scans at 1.5 T (GE 1.5 Sigma Twin Speed) of the mid-thigh and distal lower limbs were acquired; the images were representative of the whole-muscle features. Moreover, where the muscle bulk is greater, muscle involvement can be more clearly analyzed. The following skeletal muscles were assessed: gluteus maximus, anterior compartment of the thigh (rectus femoris, vastus lateralis, intermedius, and medialis), posterior compartment of the thigh (sartorius, gracilis, adductor longus, adductor magnus, semimembranosus, semitendinosus, and the long and short heads of biceps femoris), anterior compartment of the distal lower limbs (tibialis anterior, extensor digitorum, peroneus brevis, and peroneus longus), deep posterior leg compartment (tibialis posterior, popliteus, flexor digitorum, and hallucis), and superficial posterior leg compartment (gastrocnemius and soleus). The extensor hallucis and digitorum longus were evaluated as one muscle because they could not be reliably differentiated from one another in all of the scans.

The areas of signal hyperintensity in the T1W sequences were interpreted as areas of fatty infiltration. The extent of fatty replacement and its distribution in muscles were evaluated by staging, with specific scores being assigned according to the modified Mercuri scale [16]. The scans were examined by two independent observers (J.X. and J.D.), who were blinded to the clinical data, to identify abnormal muscle bulk and signal intensity within different muscles. When different scores were assigned to the proximal and distal images, the average score was used.

### Muscle pathology

Open-muscle biopsies were performed on all patients. For histological examination, serial frozen sections (8 μm) were stained by routine histochemistry, including hematoxylin-eosin, modified Gomori trichrome, Oil Red O, periodic acid Schiff, succinate dehydrogenase, nicotinamide adenine dinucleotide tetrazolium reductase (NADH-TR), cytochrome *c* oxidase, and muscle fiber ATPase at varying pH levels. For electron microscopic examination, muscle samples were fixed in 2.5% glutaraldehyde then in 1% buffered osmium tetroxide, dehydrated in ascending grades of ethanol, and embedded in Epon resin. Semi-thin sections of muscle were cut and stained with toluidine blue. Thin sections were double-stained with uranyl acetate and lead citrate.

### Molecular genetics

All patients were analyzed for (GCN) expansion in exon 1 of *PABPN1* by polymerase chain reaction (PCR) followed by direct sequencing to exclude OPMD [17]. The CTG repeat in the 3′ region of the *DMPK* gene, which is causative for DM1, was assessed by triplet repeat primed PCR. Targeted next-generation sequencing of 142 nuclear genes associated with 197 types of muscular disorders was performed using Nextera kits (Illumina), including genes for all known muscular dystrophies, congenital myopathies, distal myopathies, vacuolar myopathies, and myofibrillar myopathies (S1 Table).

## Results

### Clinical data

**Fig 1:**
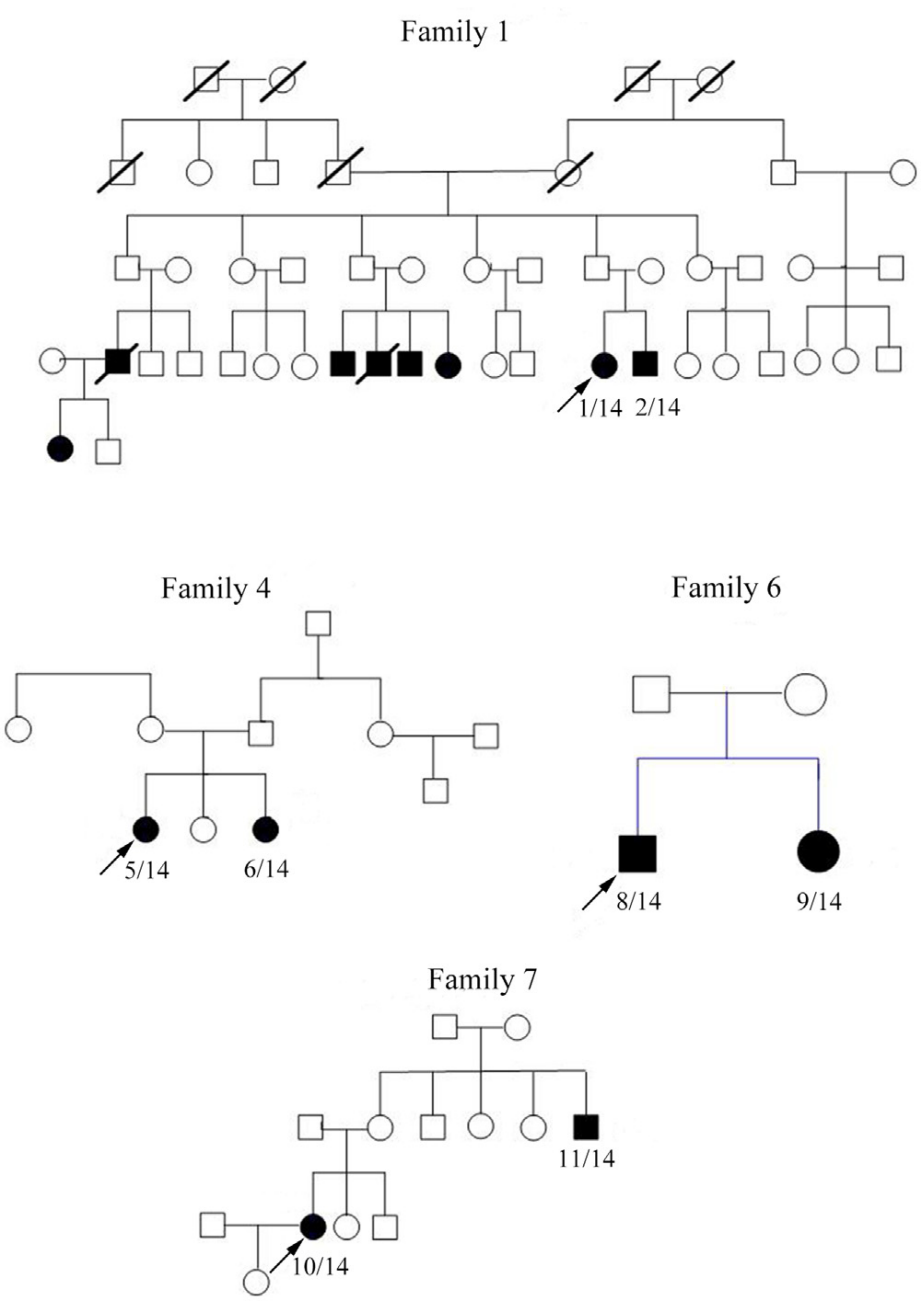
Pedigrees of the four OPDM families included in our study.

As indicated in Table 1 and Fig 1, autosomal recessive inheritance was suggested in two OPDM families: Family 1 appeared to exhibit incomplete autosomal dominant inheritance, whereas Family 7 was compatible with either X-linked or autosomal recessive inheritance. The remaining six patients exhibited sporadic or autosomal recessive inheritance.

**Table 1:** Summary of clinical manifestations and laboratory examinations results in 14 patients with OPDM.

Patient No.	Sex	Age at onset(y)	Age at imaging(y)	Initial symptom	Distribution of extremity weakness	CK^#^ (IU/L)	EMG pattern
1	F	14	32	Weakness in closing eyes	Distal > proximal	849	Myopathic change& myotonic discharges
2	M	14	32	Weakness in closing eyes	Distal > proximal	1211	Myopathic change
3	M	29	39/40	Difficulty in standing up	Distal > proximal	353	Neurogenic change
4	M	34	42	Nasal speech	Distal > proximal	190	Myopathic change
5	F	19	28	Weakness in bilateral legs	Distal > proximal	507	Myopathic change
6	F	18	24	Weakness in climbing up stairs	Distal > proximal	600	Myopathic change
7	F	36	44	Weakness in climbing up stairs	Distal > proximal	174	Myopathic change
8	M	30	35	Weakness in bilateral legs	Distal > proximal	NA	Myopathic change &myotonic discharges
9	F	26	36	Bilateral ptosis	Distal > proximal	NA	NA
10	F	27	34	Bilateral ptosis and restricted eyes movements	Distal > proximal(only in upper limbs)	146	Normal
11	M	35	42	Bilateral ptosis and restricted eyes movements	Distal > proximal	NA	Myopathic change
12	M	26	29/30	Weakness in climbing up stairs	Distal > proximal	1211	Myopathic change
13	M	20	24/26	Weakness in the right leg	Distal > proximal	1090	Myopathic change &myotonic discharges
14	F	26	30	Bilateral ptosis	Distal > proximal	581	Myopathic change

^#^ Normal limits: 70–170 IU/L; NA: data not available

The median age of disease onset was 25.3 ± 7.3 years (range, 14–36 years). Initial complaints included lower leg weakness in seven patients, eye muscle weakness during closing in two patients, ptosis in four patients, and nasal speech in one patient. The complete OPDM phenotype developed in all patients after a disease duration of 2–14 years. Slightly droopy eyelids occurred in 11 patients, and partial or total ophthalmoplegia was observed in 10 patients. Facial muscle wasting was evident in 10 patients, with the orbicularis oculi, malar and zygomatic muscles predominantly affected. Dysphagia and a hoarse or nasal voice occurred in all patients after a disease duration of up to 2 years. All patients exhibited limb weakness, with the distal muscles predominantly affected.

### Laboratory examination

The electrocardiogram and ultrasonic cardiogram results were normal for all 13 patients who underwent this exam. Serum creatine kinase (CK) levels were between 174–1,211 IU/L (normal, 70–170 IU/L). Electromyography (EMG) revealed myogenic changes in nine out of 11 patients, neurogenic changes in one, and normal findings in one patient. Myotonic discharge was observed in three patients. Fiber optic endoscopic examination of swallowing in six patients revealed both impaired velopharyngeal competency with weak elevation of the soft palate and weakness of pharyngeal muscles, whereas movement of the vocal cords was normal.

Respiratory function evaluation indicated that FVC was normal in five patients (85.1–114.8% of predicted, mean 95.38 ± 10.12%) and decreased in another five patients (52.6–76.6% of predicted, mean 69.04 ± 11.70%). FEV1 was normal in six patients (84.4–104.2% of predicted, mean 90.37 ± 7.00%) and reduced in four (51–74.4% of predicted, mean 63.75 ± 10.80%). The FEV1 and FVC values in the supine position were less than those in the sitting position in four and three patients, respectively. The FEV1/FVC ratio was normal in all patients both in the sitting (79.3–118.1%, mean 99.26 ± 11.25%) and supine positions (90–94.3%, mean 91.8 ± 1.80%). Eight patients exhibited decreased MIP and MEP, whereas two patients exhibited normal MIP.

### Muscle MRI findings

**Fig 2:**
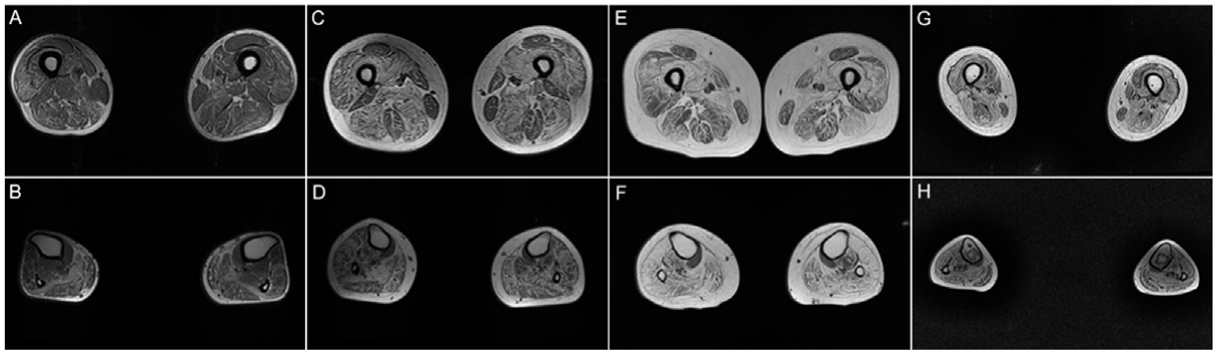
Axial T1-weighted MRI images for patients with different severities. (A) and (B) patient 13: mild weakness, ambulatory without support and with a disease duration of 6 years. (C) and (D) patient 12: mild weakness, ambulatory without support and with a disease duration of 4 years. (E) and (F) patient 7: moderate weakness, ambulatory without support and with a disease duration of 8 years. (G) and (H) patient 5: severe weakness, nonambulatory and with a disease duration of 9 years. In the early stages of the disease, low levels of fatty replacement appeared in the quadriceps, and the soleus exhibited serious involvement. With disease progression, severe fatty replacement was observed in both the thigh and lower limb muscles. Severe fatty replacement and atrophy developed during the later stages, although the vastus gracilis and short head of the biceps femoris were relatively spared.

**Fig 3:**
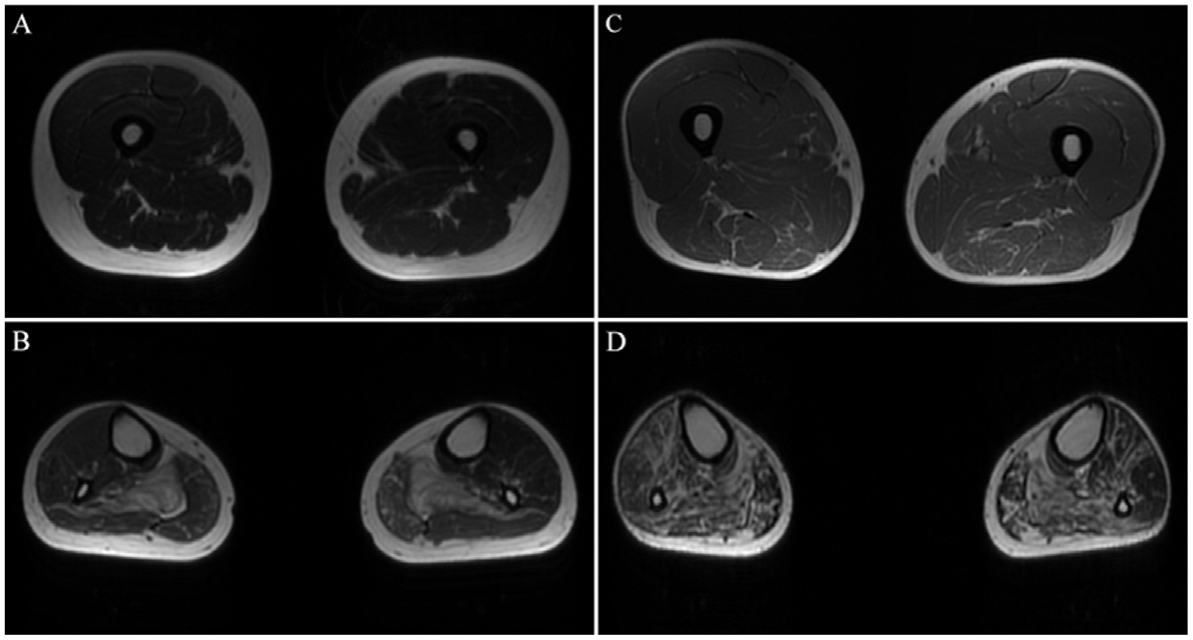
Axial T1-weighted MR images of patient 10 and patient 11. (A) and (B), Patient 10; (C) and (D), Patient 11. Both patients exhibited serious fatty replacement in the lower limb muscles, with particular involvement of the soleus. Only mild abnormalities were noticed in the quadriceps of patient 11.

**Fig 4:**
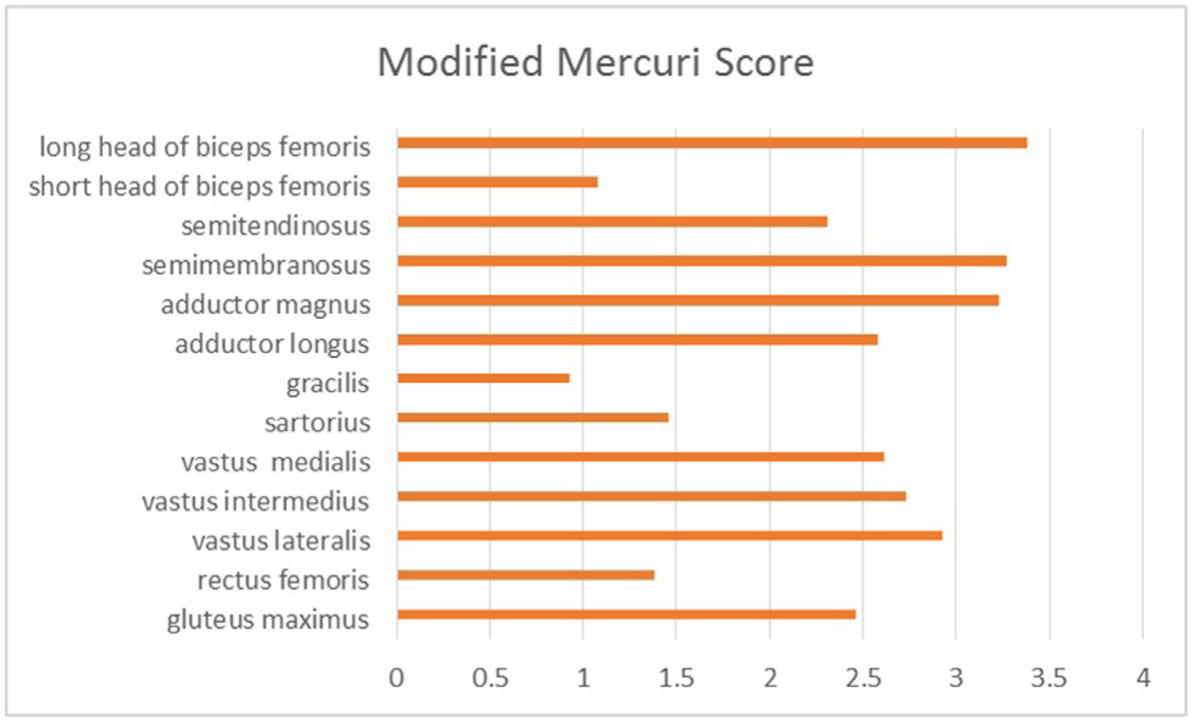
The average modified Mercuri score of the thigh muscles in 10 patients with OPDM. All thigh muscles could be involved to different extents, with the short head of the biceps femoris and the gracilis being least involved.

**Fig 5:**
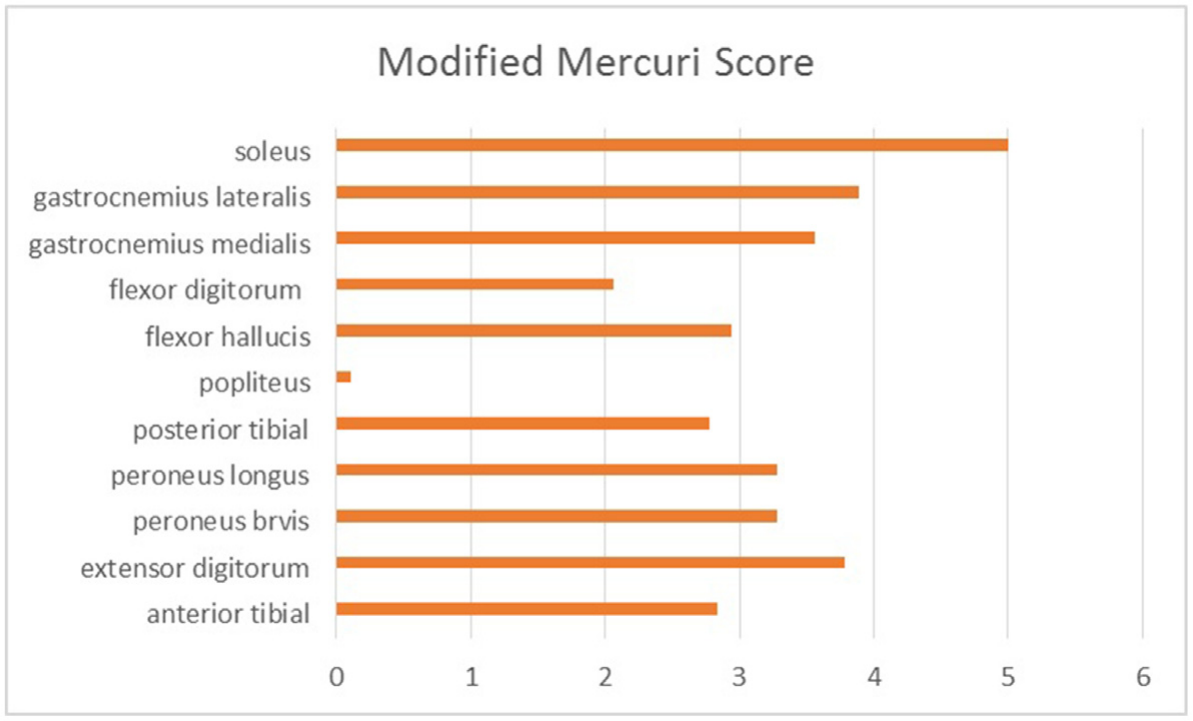
The average modified Mercuri score for the lower leg muscles in 10 patients with OPDM. All lower leg muscles were involved. The soleus, gastrocnemius lateralis and extensor digitorum were the most frequently and seriously involved, whereas the popliteus was the least involved.

A total of 10 patients underwent muscle MRI examinations. Muscle imaging findings indicated mild to severe fatty replacement (Figs 2 and 3). The lower leg muscles were consistently more seriously affected than the thigh muscles. At the thigh level, the long head of the biceps femoris was the most seriously affected by fatty replacement (the average modified Mercuri score was mMs = 3.38), followed by the semimembranosus (mMs = 3.27) and adductor magnus (mMs = 3.23). The gracilis (mMs = 0.92) and short head of the biceps femoris (mMs = 1.08) were the least involved, followed by the rectus femoris (mMs = 1.38) and sartorius (mMs = 1.46). Quadriceps femoris involvement was demonstrated in eight patients, five of whom exhibited serious fatty replacement in the vastus lateralis, vastus intermedius, or vastus medialis (mMs≥4). Another two patients exhibited no abnormalities in the quadriceps femoris (mMs = 0) (Fig 4). At the lower leg level, the soleus exhibited the most serious fatty replacement, with a score of 5 in all patients, followed by the gastrocnemius lateralis, extensor digitorum (mMs = 3.80), and gastrocnemius medialis (mMs = 3.60). The peroneus brevis and peroneus longus were equally affected (mMs = 3.28), exceeding the involvement of the tibialis anterior, tibialis posterior, flexor hallucis, and flexor digitorum. Overall, the deep posterior leg compartment was the least involved in all patients, with the popliteus completely spared in nine patients (mMs = 0), and only low levels of fatty replacement were observed in another patient (mMs = 1) (Fig 5). Most patients exhibited symmetric abnormalities, but three exhibited asymmetric involvement.

For the three patients who underwent thigh muscle MRIs twice, the imaging data revealed that fatty replacement became more serious in the bilateral vastus lateralis in cases 3 and 13, whereas case 12 exhibited more obvious fatty changes in the bilateral vastus lateralis, vastus intermedius, and vastus medialis.

As the disease progressed, different muscles became involved. The soleus, extensor digitorum, and long head of the biceps femoris were found to be seriously compromised from the early stages of the disease, whereas the rectus femoris, sartorius, gracilis, short head of the biceps femoris, and popliteus were never or only mildly involved even in the late stages of the disease. The adductor magnus and semimembranosus exhibited significant involvement 6 years after disease onset. The vastus lateralis, intermedius, and medialis, adductor longus, tibialis anterior, peroneus brevis, peroneus longus, and gastrocnemius were seriously involved after a disease duration of 7 years. After a long disease duration, the semitendinosus, tibialis posterior, flexor halluces, and flexor digitorum were mildly to moderately involved, and significant fatty replacement had developed.

### Muscle pathological findings

**Fig 6:**
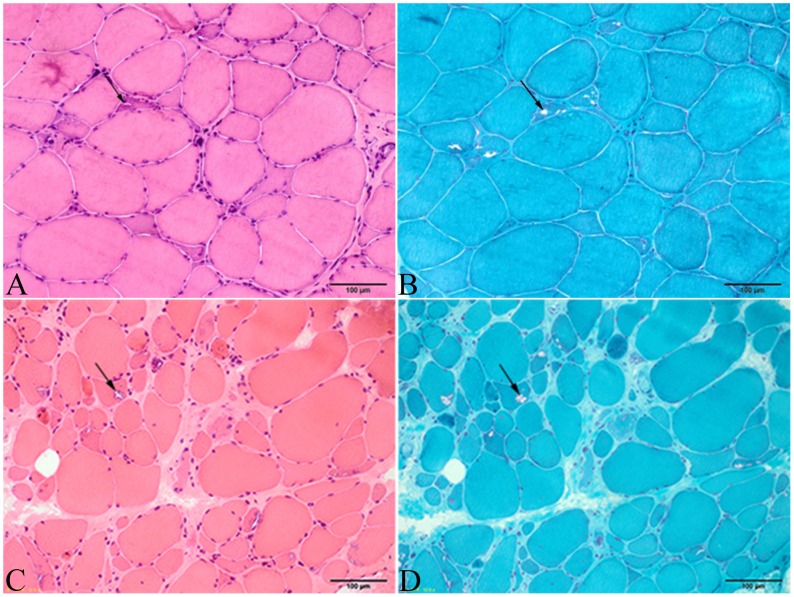
Myopathological changes in OPDM patients with various disease durations. (A) and (B) For patient 13, muscle biopsies exhibited myopathic features, including fiber size variation and rimmed vacuoles (arrowed). (C) and (D) For patient 6, muscle biopsies revealed rimmed vacuoles (arrowed), along with marked muscular dystrophic changes, including fiber size variation and endomysial proliferation (Bar = 100 μm).

**Fig 7:**
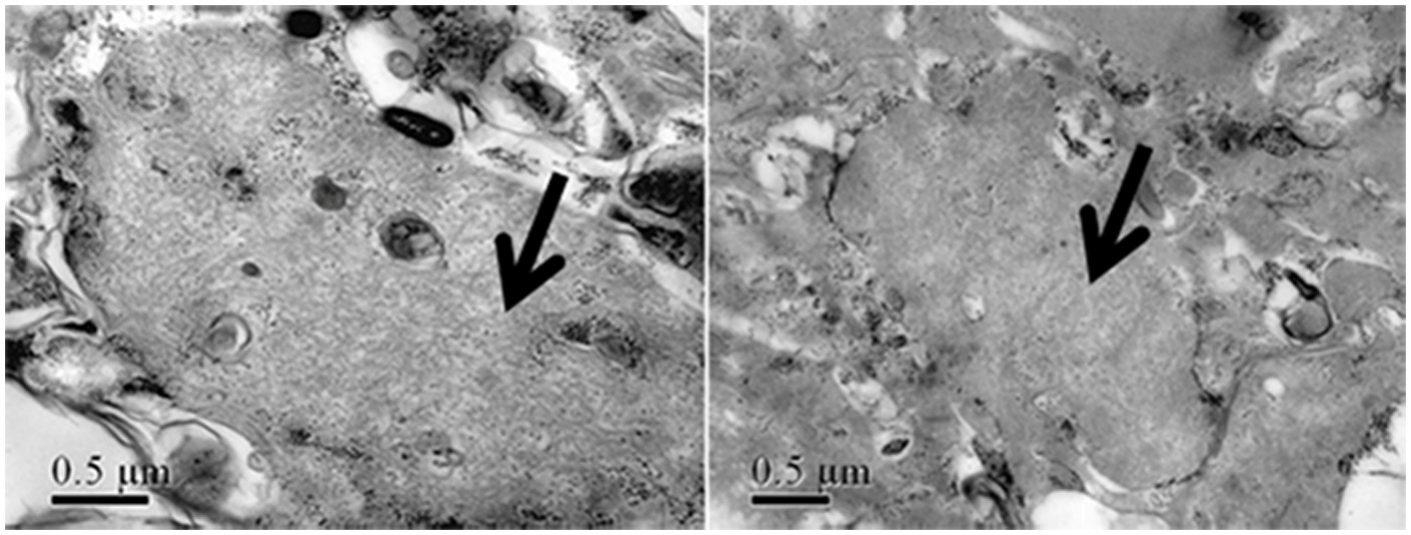
Electron microscopy demonstrated tubulofilamentous inclusion bodies in the cytoplasm (arrowheads). Left: Patient 3, biopsy from the left biceps brachii. Right: Patient 5, biopsy from the right biceps brachii. (Bar = 0.5 μm).

Skeletal muscle biopsies performed in 14 patients in the early stages of the disease only revealed myopathic changes with fiber size variation and rimmed vacuoles in all patients, and varying degrees of connective tissue proliferation, which represented muscular dystrophic changes, were observed, especially in the late stage of the disease (Fig 6). Muscle fiber necrosis and regeneration were not obvious. Electron microscopy indicated the presence of rimmed vacuoles filled with myelin figures and tubulofilamentous inclusions that measured 16–18 mm in all patients (Fig 7).

### Molecular genetic studies

All patients were demonstrated to have a wild type (GCG)_6_ repeat in exon 1 of the *PABPN1* gene by direct sequencing of PCR products. Triplet repeat primed PCR revealed that none of our patients had a repeat length that exceeded 50 CTG repeats in the 3′ region of the *DMPK* gene. No causative mutation in the 142 nuclear genes associated with muscle diseases was identified by targeted next-generation sequencing.

## Discussion

Cases of OPDM have previously been reported in Turkey, Japan, Italy, the Netherlands, and the United States [1,2,5–7]. Additionally, several Chinese OPDM patients have been reported by us and others [3,6,9]. In the present study, we reported a comprehensive clinical and molecular genetic investigation of a cohort of mainland Chinese patients with OPDM. All known muscular dystrophies or myopathies with clinical features similar to those of OPDM were excluded by PCR or targeted next-generation sequencing in these patients.

Our patients exhibited a non-uniform pattern of inheritance, although autosomal recessive or sporadic inheritance appeared to predominate. The median age of onset (25.3 years) was similar to that reported in OPDM patients from Turkey and the Netherlands [1,7], although previous Turkish patients initially exhibited bilateral ptosis as the dominant symptom, which differed from the main complaint of lower leg weakness reported by our patients [5]. Our study also demonstrated a tendency for patients that initially presented with bilateral ophthalmoplegia to exhibit a relatively slower disease progression than those with initial limb weakness, as observed in the Turkish patients. Notably, facial muscle wasting was another common feature of our patients. This feature has rarely been observed in association with OPDM, other than by Durmus et al. [1].

Fiber optic endoscopic examination of swallowing was performed to explain the observed dysarthria and dysphagia in OPDM. It revealed velopharyngeal incompetency with weak elevation of the soft palate and impaired pharyngeal muscles contraction, which may contribute to bulbar paralysis; however, movement of the vocal cords was normal. Durmus et al. previously reported myopathic changes in the thyroarytenoid and cricopharyngeal sphincter muscles, along with disproportional bowing of the vocal cords in association with OPDM [1], thus suggesting that more cases and more elaborate examinations are needed to assess the incidence of these myopathic changes in further studies.

We observed that respiratory muscles were often affected in our OPDM patients, even in those with a disease duration of only 3 years, which was less than the disease duration at which others have observed respiratory muscle involvement in affected Turkish patients [1]. Another common finding, decreased FEV1/FVC and FVC values but normal FEV1/FVC ratios, was observed in about half of the patients that underwent respiratory function evaluation, thereby indicating that restrictive ventilatory impairment was typical of these patients with early limb weakness. Significantly decreased MIP and MEP also indicated the presence of impaired inspiratory and expiratory muscle functions. However, none of these patients had presented with complaints of dyspnea to date, and no significant postural drop of FVC from the supine to sitting position was observed in our patients. This discrepancy could be explained by underlying wasting of the facial muscles. Importantly, it suggests that attention should be paid to the respiratory function of OPDM patients and that mechanical ventilation could be considered in cases of respiratory failure.

To the best of our knowledge, this study is the first to summarize the imaging patterns of OPDM and to present imaging characteristics that are particular to this disease. Overall, the distal lower legs were more significantly involved than the thigh muscles in terms of fatty replacement, which indicates consistency between the clinical presentations and imaging findings. Serious involvement of the soleus and long head of the biceps femoris was observed in all patients. The popliteus was completely spared from disease, whereas the rectus femoris, sartorius, gracilis, and short head of the biceps femoris were only mildly involved, regardless of the disease duration. This result differs from the finding that the rectus femoris and adductor magnus muscles became mildly involved in the eleventh year of OPMD disease progression in an Italian patient [5]. Moreover, we found that the soleus, gastrocnemius and extensor digitorum were significantly involved from the early stages, which was different from the observation of particularly extensive fatty replacement of the gastrocnemius medialis observed by Mignarri et al. [5]. The tibialis anterior exhibited variable signs of fatty infiltration between patients. The impairment of plantar flexion caused by involvement of the soleus was partially compensated for by a relatively milder involvement of the deep posterior leg compartment. Other muscles of the thigh and lower legs became gradually involved as the disease progressed.

Recent muscle MRI studies of neuromuscular disorders have demonstrated consistent patterns of muscle involvement in most patients who harbor mutations in a given gene [10,13–16,18–24]. Therefore, muscle imaging characteristics may offer valuable information for differential diagnosis, regardless of the clinical similarity or variability. Muscle MRI examination is particularly important for OPDM because the causative gene is still unknown. First, as an endophenotype marker, muscle pattern recognition can help physicians identify identical phenotypes in OPDM patients, which can aid further genetic studies. Second, muscle MRI can help differentiate other myopathies that have a distribution of muscle weakness and histopathological findings that are similar to those of OPDM. For example, in OPMD, the prominent imaging pattern has been reported to be involvement of the adductor and hamstring muscles in the thigh and the soleus and gastrocnemius muscles in the lower leg [19,20]. In OPDM, however, the short head of biceps femoris was almost completely spared, even in the advanced stages. DM1, another disease characterized by facial and distal limb muscle weakness with myotonic discharge, most frequently results in severe gastrocnemius medialis and soleus pathology, similar to OPDM. However, the involvement of the tibialis posterior muscle exhibits differences for these two diseases: this muscle is often affected in OPDM but relatively spared in DM1 [21,22]. Sometimes GNE myopathy can be misdiagnosed as OPDM because both can result in soleus and gastrocnemius medialis dysfunction, even in the early stages [15,23]. However, the tibialis anterior involvement and vastus lateralis spared in GNE myopathy, and the long head of the biceps femoris involvement and popliteus and the short head of biceps femoris relatively spared in OPDM, can allow these diseases to be differentiated. *MATR3* mutations associated with vocal cord and pharyngeal distal myopathy can present with bulbar symptoms and late-onset distal weakness, but muscle MRIs clearly indicate more fatty degenerative changes occurring in the anterior compartment calf muscles than in the posterior compartment [24]. Myofibrillar myopathy is often characterized by weakness and atrophy of the distal lower limb muscle, with onset in early adulthood. Muscle biopsies can reveal autophagic rimmed vacuoles and tubulofilamentous inclusions. However, in patients with desminopathy, the semitendinosus is affected at least as much as the biceps femoris, whereas the peroneal muscles are never less involved than the tibialis muscle; in most patients with myotilinopathy, the adductor magus exhibits more alterations than the gracilis, and the sartorius muscle is at least as affected as the semitendinosus; in filaminopathy, the biceps femoris and semitendinosus are at least as affected as the sartorius muscle, whereas the gastrocnemius medialis is more affected than the gastrocnemius lateralis [25]. All of these defined imaging patterns differ from those of OPDM observed in the present study, thereby enabling the diseases to be distinguished to some extent using imaging data.

In conclusion, we reported 14 mainland Chinese patients with OPDM and provided a detailed clinical evaluation which demonstrated that their phenotype exceeded the spectrum of oculopharyngodistal myopathy, and was more compatible with faciooculolaryngopharyngeal myopathy with distal and respiratory involvement, as reported by Durmus et al. [1]. We described the specific MRI pattern of muscle involvement in this patient series, which may offer valuable information for differential diagnosis and provide further evidence that OPDM is indeed a unique disease phenotype.

## Supporting Information

S1 Table The list of 142 nuclear genes associated with 197 types of muscular disorders. (DOCX)
