# Supplementary material for: Clinical and Muscle Imaging Findings in 14 Mainland Chinese Patients with Oculopharyngodistal Myopathy
Source: PLoS One. 2015 Jun 3;10(6):e0128629. doi: 10.1371/journal.pone.0128629 (PMC4454561; doi:10.1371/journal.pone.0128629)
Supplement: S1 Table — (DOCX) [file pone.0128629.s001.docx]

**Supplementary Table 1 The list of 142 nuclear genes associated with 197 types of muscular disorders.**

| ABHD5 | CFL2 | ENO3 | HNRNPA1 | LARGE | PFKM | SCN4A | TK2 |
| --- | --- | --- | --- | --- | --- | --- | --- |
| ACADL | CHKB | ETFA | HNRNPA2B1 | LDB3 | PGAM2 | SCN5A | TMEM43 |
| ACADM | CLCN1 | ETFB | HNRPDL | LDHA | PGK1 | SEPN1 | TMEM5 |
| ACADS | CNTN1 | ETFDH | IKBKAP | LMNA | PGM1 | SGCA | TNNI2 |
| ACADVL | COL6A2 | FHL1 | ISPD | LPIN1 | PHKA1 | SGCB | TNNT1 |
| ACTA1 | COL6A3 | FKRP | ITGA7 | MEGF10 | PHOX2A | SGCD | TNNT3 |
| ACVR1 | CPT1A | FKTN | KBTBD13 | MTM1 | PLEC1 | SGCE | TNPO3 |
| AGL | CPT2 | FLNC | KCNA1 | MYBPC3 | PNPLA2 | SGCG | TOR1A |
| ALG13 | CRYAB | G6PC | KCNE3 | MYH2 | POLG | SGK196 | TPM2 |
| ANO5 | DAG1 | GAA | KCNH2 | MYH3 | POMGNT1 | SLC22A5 | TPM3 |
| B3GALNT2 | DES | GBE1 | KCNJ18 | MYH7 | POMT1 | SLC25A4 | TRAPPC11 |
| B3GNT1 | DNAJB6 | GDF8 | KCNJ2 | MYH8 | POMT2 | SLC37A4 | TRIM32 |
| BAG3 | DNM2 | GMPPB | KCNQ1 | MYLK2 | PRKAG2 | SPAST | TTN |
| BIN1 | DPM1 | GNE | KIF21A | MYOT | PTPLA | SUCLA2 | TTR |
| CACNA1A | DPM2 | GTDC2 | KLHL40 | NEB | PYGM | SYNE1 | TUBB3 |
| CACNA1S | DPM3 | GYG1 | KLHL9 | OPA1 | RBCK1 | SYNE2 | VCP |
| CAPN3 | DYSF | GYS1 | LAMA2 | PABPN1 | RRM2B | TCAP |  |
| CAV3 | Dystrophin | HADH | LAMP2 | PEO1 | RYR1 | TIA1 |  |
